# Combined genomic and phenomic analyses reveals multifunctionality of *Paenibacillus polymyxa* K16 for plant’s nutrition, growth and health

**DOI:** 10.1038/s41598-025-15862-4

**Published:** 2025-09-29

**Authors:** Francesco Vitali, Paweł Trzciński, Andrea Manfredini, Expedito Olimi, Gaia Bigiotti, Magdalena Ptaszek, Lidia Sas-Paszt, Samuel Bickel, Gabriele Berg, Loredana Canfora, Eligio Malusà, Stefano Mocali

**Affiliations:** 1 Research Centre for Agriculture and Environment, Council for Agricultural Research and Economics (CREA-AA), Via di Lanciola, 12/A, Firenze, 50125 Italy; 2 The National Institute of Horticultural Research, Skierniewice, 96-100 Poland; 3 Research Centre for Agriculture and Environment, Council for Agricultural Research and Economics (CREA-AA), Via della Navicella, 2–4, Roma, 00184 Italy; 4 Institute of Environmental Biotechnology, Graz University of Technology, Graz, 8010 Austria; 5 Research Centre for Viticulture and Enology, Council for Agricultural Research and Economics (CREA-VE), Via P. Micca, 35, Asti, 14100 Italy

**Keywords:** Microbial biostimulant, Microbial biological control agent, Genome mining, Phenotype microarray, Whole genome sequencing, Strain characterization, Industrial microbiology, Bacterial genomics, Environmental sciences, Environmental biotechnology, Soil microbiology

## Abstract

The application of beneficial microorganisms with biostimulant, biofertilizer, and/or biopesticide properties represents an alternative to the use of chemicals in agriculture. Nevertheless, bioproducts’ selection and application efficacy under field condition need to be improved. A deeper understanding of the bioinoculant strains at genomic and phenomic level, would advance selection process and field application. The objective of this manuscript was to develop a combined genomic-phenomic approach for the characterization of bioproducts, and to demonstrate its application with a real use-case scenario. At the genomic level, the presence of several functional genes supporting plant nutrition (e.g., nitrate conversion into ammonia, phosphorous solubilization, siderophore production), improving plant growth (e.g., plant hormones) or promoting plant health through antagonism to plant pathogens, pointed to the multifunctionality of the strain. The phenomic analysis showed various carbon, nitrogen, phosphate, and sulfur utilization patterns, and confirmed the expression of the associated metabolic pathways. Our analysis approach encompasses as many aspects of the genome and phenome as possible for understanding the complexities of a microorganism, enabling the generation of greater value compared to the individual technologies. The proposed approach unlocks data-driven decision making in bioproduct optimisation, as such should be routinely included in screening programs.

**Supplementary Information:**

The online version contains supplementary material available at 10.1038/s41598-025-15862-4.

## Introduction

Designing novel regenerative agricultural practices has become a need for modern agriculture to fulfil technical, economic, social, and regulatory requirements. Innovative practices should reduce the environmental footprint of agriculture and the food system^1^ and increase resilience against the stresses imposed by chemical inputs and climate change. The application of products based on beneficial microorganisms can be a practice fostering the achievement of these goals. Bioproducts, i.e., natural based formulated products containing beneficial microorganisms, can promote plant growth, health, and yield thanks to their biostimulant, biofertilizer, and/or biopesticide functions^2–4^. The commercial development and use of bioproducts in agriculture have increased in the last decades, also thanks to active research around the world^5^. Indeed, large-scale isolation, genome sequencing and functional screening efforts are underway in both academic and industrial settings, to identify and characterize novel bacteria and their PGP traits.

Despite these advancements and commitments, there is yet no consensus on the best procedure to select plant growth-promoting (PGP) microorganisms for the development of novel commercial bioproducts^6–8^. After isolation, candidate strains are commonly screened for multiple PGP mechanisms using standard methods^9^. Those approaches have limited potential to discover novel mechanism of PGP, and are more likely to confirm the known features. Moreover, commonly screened traits are seldom correlated with the magnitude of observed plant growth promotion, suggesting that unknown mechanisms may be involved to explain (and fully exploit) the beneficial properties and effect of bioproducts^10^. Classical approaches are therefore limited in their ability to fully exploit the multifunctional potential that characterizes environmental strains.

The adoption of a systematic profiling of the genome and phenotype of a target beneficial microorganisms would bring the characterisation of a candidate bioproduct to a multidimensional level, hence, favouring its multifunctional exploitation. Such a comprehensive approach integrates information from diverse data types that is helpful to increase the bioproduct performance. The objective of this manuscript is that of promoting a broader adoption of such procedures, and to illustrate those combined approaches by leveraging the characterisation of a bacterial candidate strain for novel bioproduct development; the *Paenibacillus polymyxa* K16^11^. This bacterial species (Gram-positive, sporulating, and facultative anaerobic) is known to express plant growth promoting features and - under certain conditions - can also increase plant resilience or tolerance against pathogens^12^. In this real-case application, we combine large-scale genomic data and high-throughput phenomic analyses. Moreover, each step in the workflow is motivated with respect to the information it may provide about the possible activities and functions of the strain in the soil, and with respect to traits aiding its survival and persistence within the soil microbial community^12^.

## Methods

### Isolation and beneficial traits of *Paenibacillus polymyxa* K16

*Paenibacillus polymyxa* K16 was originally isolated from the roots of tomato plants grown in a field trial at the National Institute of Horticultural Research in Skierniewice (Poland).

#### Evaluation of plant growth promotion traits of P. polymyxa K16

Chitin degradation was estimated using a modified method described by Figueiredo de Vasconcellos^13^. Colloidal chitin medium^14^ in Petri dishes was spot-inoculated with bacterial biomass collected from the 48–72 h cultures and incubated at 26 °C for 168 h. The colourless ‘halo’ around the colony indicates the production of chitinases. Proteinase activity was assessed on skim milk agar (skim milk 10 g, agar 15 g, distilled water 1000 g). The medium was spot-inoculated with bacterial biomass collected from the 48–72 h cultures and incubated at 26 °C for 96 h. The colourless ‘halo’ around the colony indicates the production of proteinases. Nitrogen fixation was estimated on Burke broth (Sucrose 10 g, MgSO_4_ 0.2 g, K_2_HPO_4_ 0.8 g, KH_2_PO_4_ 0.2 g, FeCl_3_ 1.45 mg, Na_2_MoO_4_ 0.25 mg, distilled water 1000 g) by a modified method described by Ribeiro and Cardoso^15^. The test tubes with the nitrogen-free liquid medium were inoculated with the biomass collected from the 48–72 h cultures and incubated at 26 °C for 168 h. The turbidity of the medium indicated the ability to fix atmospheric nitrogen by the tested bacteria.

#### Evaluation of biocontrol properties of P. polymyxa K16

The production metabolites toxic to some bacteria and fungi was estimated by the dual culture method. For the test, the following microorganisms, from the collection of the National Institute of Horticultural Research (Skierniewice, Poland), were selected: *Fusarium sambucinum* (strain FS1), *Fusarium oxysporum* (strain WT42AA1), *Verticillium dahliae* (strain Eko_VC), *Botrytis cinerea* (strain BC1), *Pseudomonas sp.* (strains: Ps49A; Ps49G). All the tests were performed on nutrient-rich medium (Potato Dextrose Agar - PDA, Merck cat. no. 110130) and nutrient-poor medium (soil agar, composition: air-dry soil 10 g, agar 15 g, distilled water 1000 g). Antagonistic activity toward *Fusarium* spp. and *B. Cinerea* was assessed by spot inoculation of K16 and tested fungi on the edges of agar medium. For the tests regarding antagonistic activity toward *V. dahliae spp.* and *Pseudomonas spp.*, the agar media were first inoculated by spreading the suspension of *Verticillium* spores or *Pseudomonas* cells. Then the agar media were dried under the laminar flow for 20–30 min. Next, the prepared agar plates were spot-inoculated with K16 biomass collected from the 48–72 h cultures. Inoculated plates were cultivated for 3 (antagonism toward bacteria) or 14 (antagonism toward fungi) days at 26 °C. The area without growth of fungi or Pseudomonas bacteria around the K16 colony indicated the production of toxic metabolites.

### DNA extraction and whole genome sequencing (WGS)

DNA was extracted from a single colony of the *P. polymyxa* K16 plate, isolated from a 48 h-old culture growing on Tryptic soy broth (TSB) agar at 28 °C. A 10 µL loop of microbial culture was harvested and resuspended in the PowerBead Solution, following the instructions of the DNeasy PowerLyzer Soil kit (Qiagen). The DNA was subjected to qualitative evaluation by Nanodrop 1000 spectrophotometer (ThermoFisher), subsequently quantified by Varioskan spectrofluorimeter (ThermoFisher), using Quant-iT™ dsDNA high sensitivity (HS) Assay Kits (ThermoFisher). The DNA was then filtered and concentrated using 30 kDa Amicon ultra 0.5 mL centrifugal filters (Merk). The DNA was sent to IGA Technology Services (Udine, Italy) for analysis by WGS on Illumina NovaSeq 6000. After the primary bioinformatic analysis of Illumina reads, the genome was assembled with SPAdes v3.14.1^16^.

### Phenotype microarray analysis

The BIOLOG Phenotype Microarray (PM) technology^17^ was utilised to simultaneously test *P. polymyxa* K16 on PM microplates from Biolog inc. (Hayward, CA, USA), comprising 190 different assays of C-source metabolism (PM01, PM02 plates), 95 assays of N-source metabolism (PM03 plate), and 95 assays of P and S source metabolism (PM04 plate). Briefly, *P. polymyxa* K16 was grown on TSB agar and transferred into a sterile tube containing an IF-0a solution and resuspended until 98% transmittance was reached, as assessed with the Biolog turbidimeter. Then, 100 µL of the obtained suspension was dispensed into each well of PM01-04 plates, which were incubated at 30 °C in an Omnilog reader (Biolog Inc.) for 2 days with absorbance reading (490 nm) every 25 min. The data obtained were visualized using the Biolog Data Analysis software version 1.7. Phenomic data were analysed using the DuctApe software suite (version 0.18.2)^18^.

Additionally, the phenotypic profile of *P. polymyxa* K16 was also assessed with the use of GENIII plates (Biolog Inc.), which in this context, offered the possibility to simultaneously perform 23 chemical sensitivity assays, useful for evaluating ability to growth in the presence of different inhibitor or under adverse conditions. *P. polymyxa* K16 was cultivated on R2A (Merck, Darmstadt, Germany) for 48 h at 26 °C and then inoculated with the bacteria biomass suspended in the Inoculation Fluid B (Biolog Inc.), according to the manufacturer recommendations. The inoculated plate was incubated at 26 °C for 48 h and the results were read every 24 h with the use of microplates reader ELx 808 (Biotek, Santa Clara, CA, USA). The obtained results were interpreted by the Microlog3 software (version 5.2.01) with GENIII database (version 2.7.1) (Biolog Inc.).

### Profiling the volatile emission of *P. polymyxa* K16

The Volatile Organic Compounds (VOCs) produced by *P. polymyxa* K16 were analysed using headspace solid-phase microextraction gas chromatography with mass spectrometry^19,20^. Using an inoculation loop, the bacterial isolate was transferred to 10 ml slant agar containing nutrient broth in 20 mL headspace vials (Chromtech, Idstein, Germany). The culture was streaked in three parallel lines in three replicates. Following 48 h of incubation at room temperature (approximately 20 °C), the vials were sealed and incubated for an additional two hours before analysis. In addition, a sample containing only nutrient agar was used as a negative control to subtract the background signal. Solid phase microextraction was performed with an automated sampler and 50/30 µm Divinylbenzene/CarboxenTM/Polydimethylsiloxane/2 cm Stableflex/SS fibre (Supelco, Bellefonte, PA, USA). The VOCs in the headspace were equilibrated with the fibre for 30 min at 35 °C. The detection of compounds was carried out on a GC 7890 A system equipped with quadrupole mass detector MS 5975 C (Agilent Technologies, Waldbronn, Germany). Samples were run through a (5%-phenyl) methylpolysiloxane column, 30 m × 0.25 mm i.d., 0.25 μm film thickness (HP-Column: HP-5MS; Agilent Technologies, Waldbronn, Germany), followed by electron ionization (EI; 70 eV) and detection within a mass range of 25–350 amu. The inlet temperature was set to 250 °C. The GC column was kept at 40 °C for 2 min, raised to 100 °C at a rate of 5 °C/min, then to 280 °C at 10 °C/min, and finally maintained at 280 °C for 3 min. The helium flow rate was set to 1.2 mL/min. The detected VOCs were identified based on retention time and mass data using the NIST 14 reference database. The mVOC version 4.0 database^21^ was used to identify VOCs of microbial origin.

### Data analysis, genome mining and metabolic reconstruction

The web server JSpeciesWS (http://jspecies.ribohost.com/jspeciesws/) was used to calculate the Average Nucleotide Identity (ANI) of the *P. polymyxa* K16 genome and related genomes in the JSpeciesWS server collection, using Tetra Correlation Search (TCS) against their GenomesDB^22^. A total of 24 genomes contained in the GenomesDB were then selected for the analysis.

The following criteria were considered to analyse the genome of *P. polymyxa* K16 to uncover functions connected with its possible use as a bioproduct:

1. *Persistence/resilience potential*: functions including genomic signatures that could be related to resistance mechanisms toward soil biotic (e.g., competition with autochthonous microorganisms) or abiotic stresses (e.g., limited mineral status, saline stress), which ensure survival of a sufficient quantity of the inoculum in the soil.
2. *Bio-fertilizer potential*: function of genomic signatures that could be connected to the improvement of plant or soil nutritional status (e.g., N and P availability).
3. *Plant growth promoting potential*: function referring to genomic signatures that could be connected to beneficial effects on plant growth (e.g., phytohormone production).
4. *Bio-control potential*: function referring to the genomic signatures that could be connected to the production of antimicrobial compounds.

The AntiSMASH tool (version 7.0)^23^ was used to mine for the presence of known Biosynthetic Gene Clusters (BGCs). The Comprehensive Antibiotic Resistance Database (CARD) (https://card.mcmaster.ca/)^24^ was used to annotate antibiotic resistance gene clusters applying the “Perfect, Strict, and Complete genes only” criterium. The genome was annotated using the RAST online service^25^. Gene annotation tables were used to search the genome for the presence of genes associated to nitrogen and phosphorus cycles by using wildcard characters (i.e., searching for “nitr*” and “phosph*” in the subsystem and function name). Moreover, phosphorous cycle genes listed by Liang et al.^26^ were searched in the annotated genome using their KEGG ortholog code. Lastly, siderophores were searched in the genome by using the FeGenie tool^27^.

Genomic and phenomic data were used to annotate selected KEGG^28^ pathways for Nitrogen metabolism (map00910) and Sulfur metabolism (map00920). This analysis aims to combine the gene present in genomes, obtained with RAST annotation (KEGG ortholog code for the different genes), with their metabolic activity (results of activity values from the different PM plates obtained with DuctApe analysis) and to visually represent the whole pathway functioning by annotating the genes (presence/absence from RAST annotation) and compounds (colour intensity scaled on calculated AV) in selected KEGG pathway maps. The analysis was performed in R^29^ using the *pathview* package^30^ with default settings.

## Results

### Genotypic characterization of the *P. polymyxa* K16

Generic features of the genome of *P. polymyxa K16*, obtained with WGS are reported in Table 1. Average Nucleotide Identity (ANI) analysis showed a 95% similarity above cutoff with 5 genomes out of other 24 *Paenibacillus* strains present in the GenomesDB, namely *P. peoriae* HS311, *P. polymyxa* CFSAN034343, *P. polymyxa* YUPP-8, *P. polymyxa* E681, *P. polymyxa* J (Supplementary Table S1). Even though metadata annotation of those genomes in NCBI was scarce (Supplementary Table S2), not allowing many inferences, they resulted to be all isolated from agricultural soils or endophytic environments, and most were expressing plant growth promoting activities.

**Table 1:** *Paenibacillus polymyxa* K16 general genomic features.

Attributes	Value
Genome size	5,978,418
Contigs	1
G + C%	45.5%
Total genes	5794
CDS	5613
RNAs	53
tRNA	52
Accession number	PRJNA889341

As reported in the following Sect. (Secondary metabolites pathways and antibiotic resistance (persistence/resilience potential*)*–Genes related to biocontrol potential), genomic mining allowed to identify multiple genes that were associated with specific functions, connected with the possible use of *P. polymyxa* K16 as a bioproduct.

#### Secondary metabolites pathways and antibiotic resistance (persistence/resilience potential)

AntiSMASH analysis identified 15 gene clusters related to secondary metabolite pathways, 7 of which were similar to known clusters (Table 2). Four of these showed 100% similarity with clusters from other genomes: fusaricidin B gene cluster, polymyxin B gene cluster, tridecaptin gene cluster, and paenalin gene cluster. Noteworthy, AntiSMASH analysis pointed out the presence of a phosphonate cluster and a siderophore cluster not presenting any degree of similarity with other *P. polymyxa* strains present in the database. The CARD tool identified 7 “strict” hits, but no “perfect” hits (Table 3). The maximum identity value (85%) concerned the complete representative sequence for the resistance to the lincosamide antibiotics through a mechanism of target alteration.

**Table 2:** Gene clusters associated with secondary metabolites production identified in *P. polymyxa* K16 using antismash. Nucleotide positions within the genome are reported. Abbreviation used: NRPS, Non-ribosomal peptide synthetase; T3PKS, type III polyketide synthase; T1PKS, type I polyketide synthase.

*n*°	Gene cluster	From (nt)	To (nt)	Most similar known cluster	Similarity
1	PRE-containing	486,310	505,872		
2	NRPS	7551,076	813,652	Fusaricidin B	100%
3	Phosphonate	999,609	1,031,728		
4	NRPS	1,655,609	1,728,892	Polymyxin B	100%
5	T3PKS, transAT-PKS, NRPS	3,073,104	3,173,353	Aurantinin B/C/D	35%
6	Cyclin-lactone-autoinducer	3,691,073	3,711,294		
7	T1PKS, NRPS	3,898,768	3,992,603	Gramicidin S	6%
8	NRPS	4,229,946	4,322,819	tridecaptin	100%
9	Cyclic-lactone-autoinducer	4,659,425	4,680,068		
10	NRPS-like	4,686,536	4,729,778		
11	Lenthipeptide-class-i	5,041,741	5,068,747	paenilan	100%
12	lassopeptide	5,397,383	5,421,498	paeninodin	40%
13	NRPS, transAT-PKS	5,456,675	5,556,654		
14	proteusin	5,607,403	5,627,639		
15	NI-independent-siderophore	5,794,941	5,812,342		

**Table 3:** Antibiotic resistance genes identified in the genome of *Paenibacillus polymyxa* K16. Abbreviation used: AMR, antimicrobial resistance; ARO, antibiotic-resistant ontology.

*n*°	AMR gene family	ARO Term	Drug Class	Mechanism	identity %	% length of reference sequence
1	Llm 23 S ribosomal RNA methyltransferase	LlmA 23 S ribosomal RNA methyltransferase	lincosamide antibiotic	antibiotic target alteration	84.67	100
2	fosfomycin thiol transferase	FosBx1	phosphonic acid antibiotic	antibiotic inactivation	65.69	101.45
3	small multidrug resistance (SMR) antibiotic efflux pump	qacG	disinfecting agents and antiseptics	antibiotic efflux	60.75	114.95
4	vanW, glycopeptide resistance gene cluster	vanW gene in vanI cluster	glycopeptide antibiotic	antibiotic target alteration	40.91	126.54
5	glycopeptide resistance gene cluster, vanT	vanT gene in vanG cluster	glycopeptide antibiotic	antibiotic target alteration	34.59	55.90
6	tetracycline-resistant ribosomal protection protein	tet (36)	tetracycline antibiotic	antibiotic target protection	31.33	102.19
7	vanY, glycopeptide resistance gene cluster	vanY gene in vanB cluster	glycopeptide antibiotic	antibiotic target alteration	30.6	112.31

#### Nitrogen and phosphorous cycles, and siderophores synthesis (bio-fertilizer potential)

A total of 21 features connected to nitrogen metabolism were found in the genome (Supplementary Table S3). Eleven of them were classified into three subsystems: the “denitrifying reductase gene clusters” (4 features), the “nitrate and nitrite ammonification” (6 features), and the “ammonia assimilation” (1 feature). The other 10 features were not classified under any subsystem. All four subunits of the respiratory nitrate reductase enzyme were identified, as well as six nitrite reductase subunits.

The RAST analysis of the *P. polymyxa* K16 genome showed the presence of several genes associated with phosphorous solubilization from inorganic (Exopolyphosphatase, *ppx;* Manganese-dependent inorganic pyrophosphatase; Inorganic pyrophosphatase, *PpaX*) and organic (Alkaline phosphatase, *PhaA*) sources. Multiple genes for phosphate transport and regulation were also identified (Supplementary Table S4).

Thirteen genes for siderophore synthesis, distributed in 5 different families (PchC, VabF, PchH, PvsD, and PvsE) were identified (Supplementary Table S5). In addition, numerous genes associated to siderophore, or iron functions were also detected: 51 genes with iron regulatory functions, 17 genes with iron acquisition and transport functions, and 44 genes with the function of siderophores transport.

#### Plant hormones (PGP potential)

The list of hormones reported in the “plant hormone signal transduction” map in the KEGG database (map04075) was utilised searching the genome annotation for the related genes.

- *Ethylene*: *P. polymyxa* K16 did not possess the necessary enzymes to produce ethylene following the pathway for “cysteine and methionine metabolism” (KEGG map00270) nor those responsible for ACC deaminase pathway. Nevertheless, ethylene could be produced from the “Chloroalkane and chloroalkene degradation” pathway (map00625) from acetylene through a nitrogenase (Enzyme 1.18.6.1 in reaction R05496 in KEGG), which was encoded in the *P. polymyxa* K16 genome.
- *Auxin*: The *P. polymyxa* K16 genome encoded an amidase (Enzyme 3.5.1.4 in reaction R03096 in KEGG) able to produce indole acetic acid from indole-3-acetamide following the pathway for “tryptophan metabolism” (KEGG map00380). Additionally, RAST annotation revealed the presence of 4 coding DNA sequences classified under the auxin biosynthesis subsystem (Supplementary Table S6).

#### Genes related to biocontrol potential

Three features in the *P. polymyxa* K16 genome were connected to chitin degradation, which may indicate biocontrol potential. Two of them were related to the chitinase function, while one was a chitinase binding function (Supplementary Table S6).

### Phenotypic characterization of the *Paenibacillus polymyxa* K16

#### In vitro phenotypic characterization

Results of in vitro phenotypic assays (Table 4) showed that the strain has potential to promote plant growth (i.e. through N-fixing capacity, and production of siderophores), as well as to favour plant protection (i.e. through production of secondary metabolites and toxins inhibiting the growth of different soil-borne pathogens - *Fusarium sp.*, or *Verticillium dahliae* - and Gram-negative bacteria - *Pseudomonas sp*). *P. polymyxa* K16 was able to produce proteases, organic acids from glycerol/sucrose/starch, and levan from starch.

**Table 4:** In vitro phenotypic characteristics of *P. polymyxa* K16 strain.

Plant Growth promotion abilities	
Chitin degradation	+
Proteinase activity	+
Nitrogen fixation	+
Growth inhibition of soil-borne pathogens	
*Fusarium spp*	+
*Verticillium dahiae*	+
*Botrytis cinerea*	+
*Pseudomonas* spp	+

Supplementary Table S7 presents result from overall high-throughput phenotyping evaluation of *P. polymyxa* K16 activity/resistance (+) or non-activity/susceptibility (-) toward 96 compounds in the GENIII plate. In summary, *P. polymyxa* K16 was able to grow under some harsh conditions: slightly acidic pH (pH 5, 6), in presence of salt (NaCl) concentrations up to 4%, and of sodium lactate, a food preservative with bacteriostatic action, at the concentration of 1% (Table 5). In addition, growth was observed in the presence of various other compounds (guanidine HCl, tetrazolium violet, lithium chloride, potassium tellurite, sodium butyrate, and sodium bromate). However, *P. polymyxa* K16 did not show antibiotic resistance, being sensitive to all the antibiotics present in the micro-assay plate.

**Table 5:** Qualitative characterization of the susceptibility/resistance profile of *P. polymyxa* K16 strain from GENIII plate micro-array. Bold font denotes resistance to the substance (+) while normal font denotes susceptibility (-).

Position in column			
	10	11	12
A	**Positive Control**	**PH 6**	**PH 5**
B	**1% NaCl**	**4% NaCl**	8% NaCl
C	**1% Sodium Lactate**	Fusidic Acid	D-Serin
D	Troleandomycin	Rifamycin SV	Minocycline
E	Lincomycin	**Guanidine HCl**	Niaproof 4
F	Vancomycin	**Tetrazolium Violet**	Tetrazolium Blue
G	Nalidixic Acid	**Lithium Chloride**	**Potassium Tellurite**
H	Aztreonam	**Sodium Butyrate**	**Sodium Bromate**

**Fig. 1:**
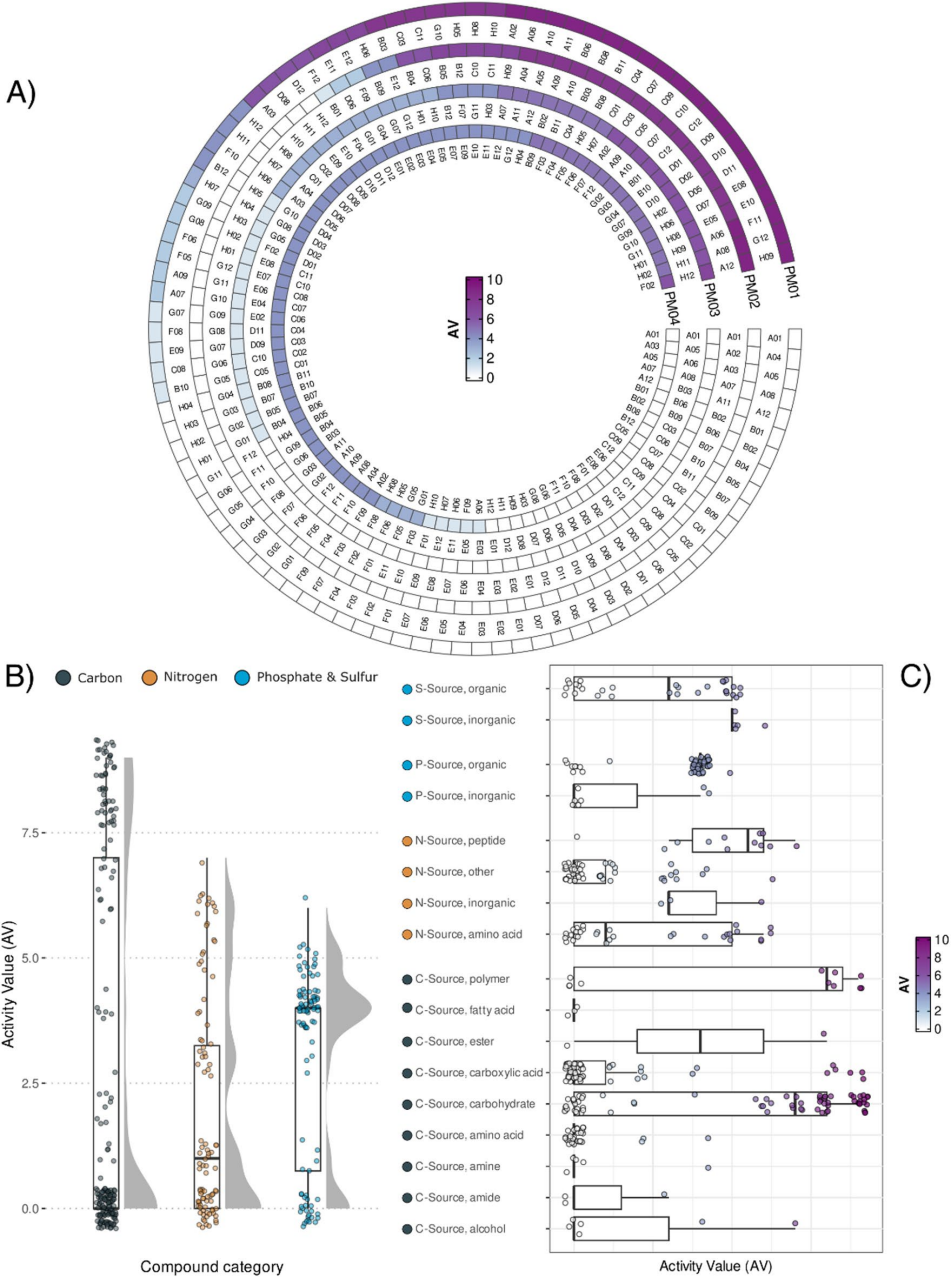
Phenomic characterization of the *Paenibacillus polymyxa* K16 strain. **A**) Activity value (AV) calculated for each well of the phenotype microarray plate used in the experiment. Each circle represents a different plate (from PM01 to PM04), and each square in the circles represent a different nutrient source (well). Labels refer to well position on the plate. **B**) Boxplot and distribution of the observed AVs obtained for each of the three categories of nutrient sources. **C**) Boxplot of the observed AVs obtained for each chemical sub-category of the three nutrient sources.

The phenomic characterization of *P. polymyxa* K16 is presented in Fig. 1. For each plate, active compounds (AV > 0) and those with no activity (AV = 0) were identified (Supplementary Table S8 and S9, respectively).

Several compound categories (carbon, nitrogen, and phosphate & sulfur) showed statistically different metabolization potential (Kruskal-Wallis test; p-value = 0.014) (Fig. 1B). However, when considering only metabolised compounds (AV > 0), those based on carbon showed a significantly higher activity value than nitrogen and phosphate & sulfur sources (Wilcoxon rank sum test; C vs. N: p-value < 0.001; C vs. P&S: p-value < 0.001), while no significant difference was found comparing the metabolic activity between the latter sources (Wilcoxon rank sum test; N vs. P&S: p-value = 0.101). The distribution of metabolic activity for C, and P&S sources, could be divided in two major groups of compounds with either high or none metabolization potential (Fig. 1B). On the other hand, such distinction was not observed with N-rich compounds, underlining a quite homogeneous metabolization capacity of N sources by *P. polymyxa* K16. Interestingly, the highest AV values and the highest sum of all AVs were recorded with C sources, though with high variability among sources; C sources had in fact the highest percentage of not-metabolised compounds (57.8% of compounds showing AV = 0) (Table 6).

**Table 6:** Summary statistics of the metabolic activity values of *P. polymyxa* K16 for the three categories of nutrient sources.

Compound Category	PM	Sum of AV	*N* of 0 AV (%)	Highest AV (*N*; %)	Median AV	Mean AV (Standard Error)	Standard Deviation	Coeff. Of Variation
Carbon	PM01 PM02	532	111 (57.81%)	9 (23; 12.00%)	0	2.77 (0.27)	3.70	1.33
Nitrogen	PM03	185	42 (43.75%)	7 (1; 1.04%)	1	1.93 (0.23)	2.26	1.17
Sulfur and Phosphorus	PM04	287	24 (25.00%)	6 (1; 1.04%)	4	2.99 (0.20)	1.93	0.65

Carbohydrates induced the highest metabolic activity among C sources (Fig. 1C) with almost half of the compounds (45%) reaching an activity value of 8 or 9, followed by polymeric compounds, with 7 out of 11 (38%) having high AV (namely Glycogen, Pectin, Dextrin, Inulin, Laminarin, b-Cyclodextrin, and g-Cyclodextrin). Among N sources, high activity (AV = 7 or 6) was only recorded for peptides and amino acids. However, the level of their metabolization was not the same: amino acids induced both high and low activity values, while high activity on ammonia was paralleled with low activity on nitrite and nitrate substrates and no activity was recorded on urea and uric acid. Regarding S sources, all inorganic sulfur sources (sulfate, thiosulfate, tetrathionate, thiophosphate, and dithiophosphate) promoted an average activity (AV = 5 or 6) while very low AV, or no activity, was recorded for 13 out of 30 (43%) organic sulfur sources. Most organic phosphate sources (83%) were actively metabolized (AV > 0), but most inorganic P sources (5 out of 7) resulted in no activity.

#### Characterization of volatile organic compounds emitted by p. polymyxa K16

**Fig. 2:**
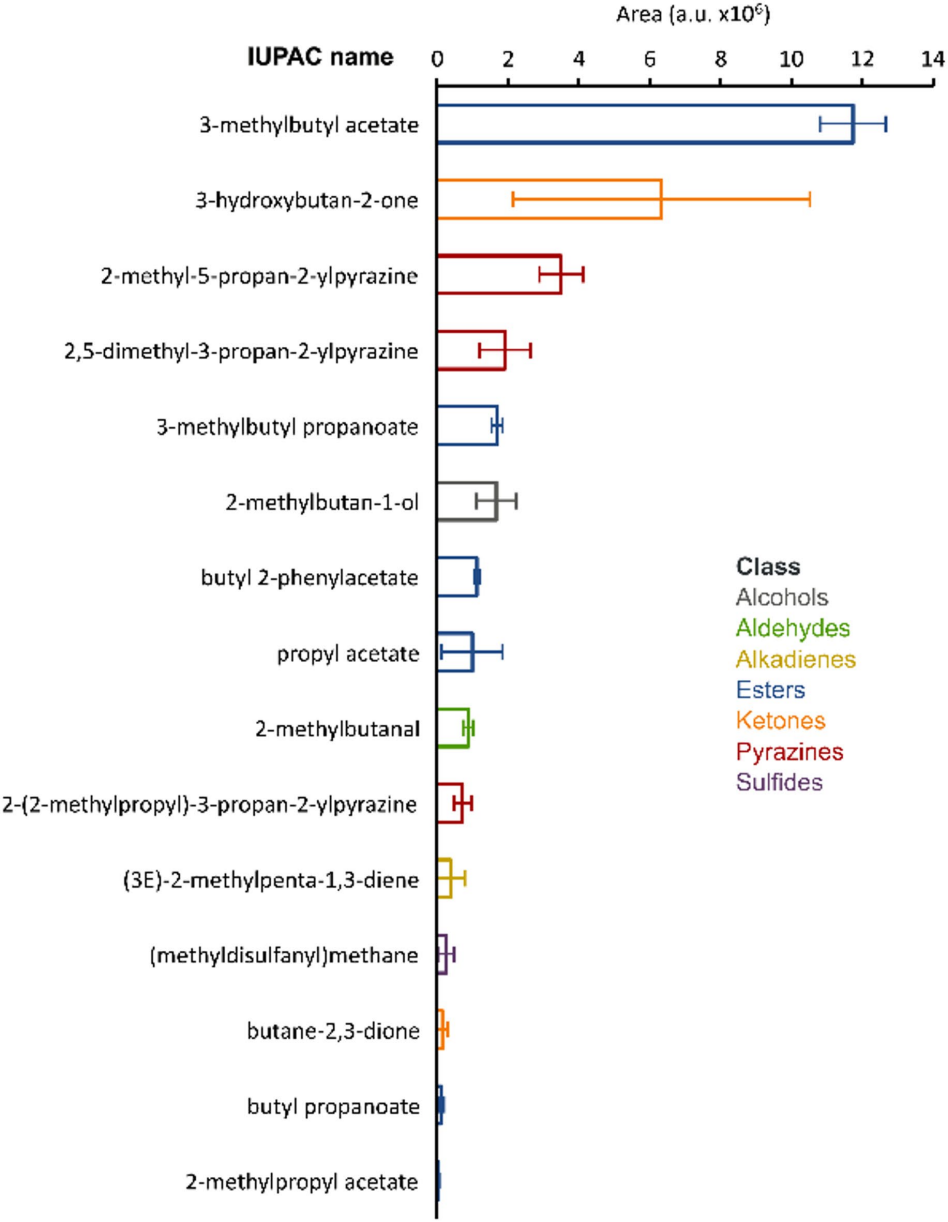
Volatile organic compounds produced by *Paenibacillus polymyxa* K16. The compounds detected by GC-MS were ranked by their peak area (mean ± SD, *n* = 3). Colours indicate the compound classes.

The VOCs produced by *P. polymyxa* K16 in vitro cultures included a variety of alcohols, esters, and pyrazines (Fig. 2). Among the 15 compounds detected, three were produced at a level significantly higher compared to the remaining one; they belonged to different chemical classes and included 3-methylbutyl acetate, 3-hydroxybutan-2-one and 2-metyl-5-propane-2ylpyrazine. All compounds except (3E)−2-methylpenta-1,3-diene were previously reported to be of microbial origin in the mVOC 4.0 database.

### Coupling phenomic characterization and genomic inferences

**Fig. 3:**
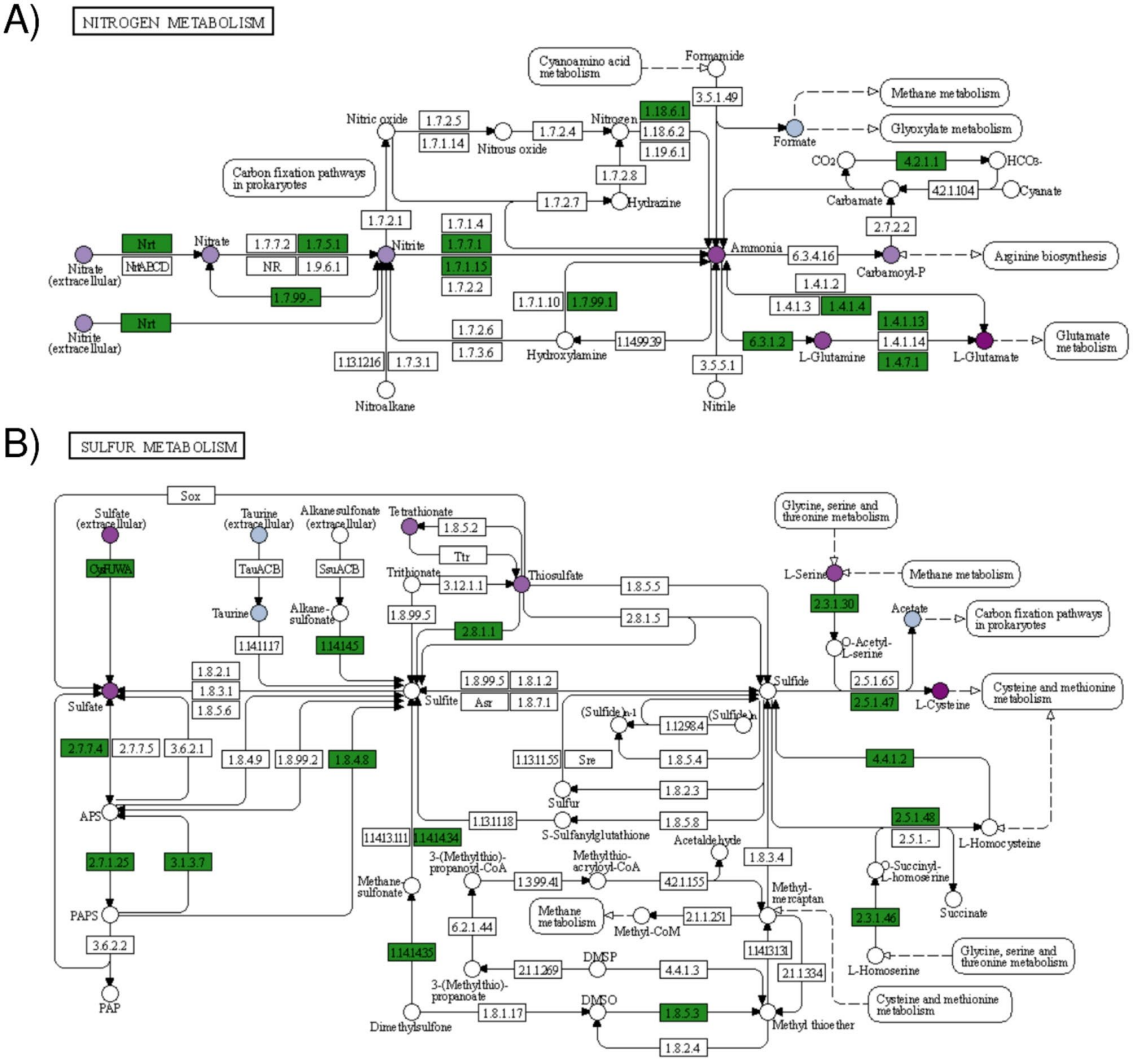
Scheme of the KEGG pathway for nitrogen metabolism. (**A**) and sulfur metabolism (**B**) with annotation of both presence/absence of the relevant gene in the genome of the *Paenibacillus polymyxa* K16 and its metabolic activity. Genes are represented by rectangles (green coloration imply its presence in K16 genome); metabolic compounds are represented by circles (darker shades of purple indicate higher AV).

To verify the coherence between genomic and phenotypic characterisation, the data from these analyses were combined annotating the scheme of the KEGG pathway for both nitrogen (map 00910) and sulfur metabolism (map 00920) with information of both presence/absence of relevant genes identified in the genome analysis and their metabolic activity (Fig. 3).

The combined analysis highlighted the consistency between the different data. In case of the N metabolism, the capacity of converting extracellular nitrate to ammonia obtained via the dissimilatory and assimilatory nitrate reduction pathways of the N metabolism was confirmed by the identification of genes for all these metabolic steps in the genome and by the analysis of metabolic activity, including ammonia (final product of the metabolic pathway) that was shown to be actively metabolized by *P. polymyxa* K16 into amino acids or other secondary metabolites (Fig. 3A).

The genomic annotations on the assimilatory sulphate reduction module in the general sulfate metabolism pathway, highlighted the ability of *P. polymyxa* K16 to reduce sulfate to sulfite, but not up to sulfide (Fig. 3B).

## Discussion

The methodological approach described in this study showed to be suitable for the characterization of the various functions expressed by a potential beneficial bacterial strain, ultimately demonstrating its multifunctionality potential.

Several genes which could support plant growth promotion through various mechanisms were observed in the genome of *P. polymyxa* K16, e.g., related to soil nutrients cycles, plant physiology, and secondary metabolic pathways increasing resilience or tolerance toward biotic stresses. These results were confirmed by both phenotypic and phenomics analyses, though highlighting a differential expression depending on the nutrient source. In addition, the genome was similar to that of other isolates from agricultural soils or endophytic environments, while differed compared to strains isolated from animal environments (gut, rumen, or insect honeycomb), pointing to a whole genome level adaptation between strains isolated from specific (micro)environmental conditions^31^. As suggested by early studies, showing a broad array of genes functional to plant growth promotion in strains of *P. Polymyxa*^32^. K16 strain possesses multiple genes endowing biofertilizers traits^26^: conversion of nitrate in ammonia, atmospheric nitrogen fixation, inorganic and organic phosphorous solubilization, and iron siderophore production. For all these traits, specific regulatory genes and transporters genes were also identified suggesting a complete metabolic pathway.

Regarding its biofertilizer potential respect to nitrogen metabolism in particular, the coupling of phenomic and genomic analyses demonstrate the activity of the genes included in the pathway, thanks to the observation of actively metabolized compounds, supporting the potential of the K16 strain to become a microbial biostimulant. Interestingly, a pangenomic analysis including strains originating from various geographical locations and environmental conditions revealed that the *nif* cluster gene associated with nitrogen fixation was rarely identified^33^, making *P. polymyxa* K16 notable with respect to this function, as it possessed various N-fixing gene clusters. In addition, results supported *P. polymyxa* K16 as a potential biostimulant with multifunctional biofertilizer traits owing to its capacity of metabolising sulfur, an element affecting soil P availability and critical for plant growth^34^.

The exploitation of PGPB in bioproducts is normally associated to a single nutrient, commonly to either N or P, while solubilization and improved availability of micronutrients is just considered an additional benefit^35^. On this regard, our results revealed the *P. polymyxa* K16 strain’s potential biofertilizers traits also respect to micronutrient^28^ owing to several genes associated with phosphorous solubilization and involved in siderophore production (which may chelate iron in soil and transfer to the plant^36^) and in iron mobilization. These evidences further confirm how a thorough genomic analysis, as presented in this paper, is pivotal to better define the bio-stimulation potential of a novel beneficial strain increasing its applicability for various purposes. Acknowledging such features in a microbial product would prompt application tailored to specific scenarios, such as in calcareous soils where iron deficiencies frequently occur, in alternative to synthetic solutions^37^.

Remarkably, our analysis pointed out the potential of the strain to indirectly enhance the plant resilience toward biotic stresses. Various BGCs encoded antimicrobial secondary metabolites and were identical to known BGCs, allowing to assume a functional biosynthesis of antimicrobials such as Polymyxin B. The observed plate inhibition activity towards filamentous fungi or bacteria confirmed such hypothesis. The gene cluster 1, having 100% similarity with the known BGCs of Fusaricidin B could be related to this inhibitory activity. Indeed, Fusaricidins^38^ provide a large reservoir of potent antifungal compounds effective in inhibiting the growth of a broad array of soil-borne phytopathogenic fungi, such as *Fusarium sp.*^39^, *Rhizoctonia solani*, and *Sclerotinia sclerotiorum*^40^. The observed toxic activity towards *Pseudomonas* could be also linked to clusters 3 and 7 (in addition to 1), which showed 100% similarity with the known BGCs of Polymyxin B and Tridecaptin, respectively, which are active against Gram- bacteria^41^. Polymyxins are a family of closely related variants cationic cyclic lipodecapeptides, and the B variant is an important molecule with therapeutic effects on human pathogens^42^.

The usefulness of the proposed approach can further be appraised considering an evaluation of a potential toxic effect of *P. polymyxa K16* toward Gram + bacteria, an in vitro test which is not routinely performed for bioproducts characterization. Genome mining predicted such potential toxic action due to the presence of gene cluster 9, having 100% similarity with the known BGCs for Paenilan^43^. Collected information on those genomic signatures could be used to effectively adapt the characterization process to the specific strain, informing on the need to include specific plate-based test as metabolic confirmation of genomic features.

Moreover, the volatile compounds produced by *P. polymyxa* play significant roles in agriculture, particularly in biocontrol and plant growth promotion. Among the VOCs produced by *P. polymyxa K16* there were several pyrazines and other compounds, which are known to both triggers Induced Systemic Resistance (ISR)^44^ and pest control activity^45–47^. For instance, 3-methylbutyl acetate has been associated with antifungal properties, which can inhibit the growth of phytopathogenic fungi, thereby enhancing plant health and resilience against diseases^47^. Additionally, compounds like 2-methyl-5-propan-2-ylpyrazine and 2,5-dimethyl-3-propan-2-ylpyrazine have been linked to VOCs that contribute to the suppression of plant pathogens^48^. Various factors (e.g. the growth substrate, environmental and ecosystem conditions) are affecting the composition of microbial VOC blends, which is also dynamically changing over time^49^. Therefore, this additional analysis provides useful information for understanding the potential of a strain.

However, further research is needed to understand how such features could be exploited in practice^50^. It is noteworthy that, despite the observed biocontrol potential of *P. polymyxa* K16 and other *P. polymyxa* strains^12^, no commercial products are registered in the European Union and USA for this use. This could indicate that the field application of *P. polymyxa* is based on its biostimulant functions and its capacity of triggering ISR, rather than directly protecting the plant against pests. The combination of genomic and phenomic characteristics can help evaluate and validate functionality for application under field conditions, but it cannot be used to infer the in situ multifunctional capacity of a strain.

Finally, the complete genome of a potentially beneficial bacterial strain can also be leveraged for assessing other aspects of bioproducts, not directly related to their biofertilizer or biopesticide activities. As an example, results highlighted that antibiotic resistance genes were poorly represented in K16 genome. Such traits are important features supporting the persistence potential of the strain when applied to the soil and to compete with soil autochthonous microbiome^35^, and would advise the manufacturer about the kind of most suitable formulation or application method that could reduce a potential competition or foster the strain persistence^51,52^. In addition, the complete genome of the strain can be used to design monitoring tools for bioproduct application, able to support the optimization of the application method of the strain formulation under various soil conditions. In this regard, it is noteworthy to mention that having the complete genome of *P. polymyxa* K16 strain allowed for designing a Taq-MAN assay for detecting and monitoring its persistence after application to soils^53^.

## Conclusion

Increasing the application of microbial biostimulants, a category of fertilising products defined by the European Union Regulation 2019/1009, is an urgent need for modern agriculture to comply with policies and consumers demands. Even though a complete substitution of agrochemical is unlikely in the short term, the optimization of their use can be fostered using microbial biostimulants, potentially leading to the effective net reduction of chemicals used. The genomic and phenomic data collected for *Paenibacillus polymyxa* K16 presented in this study contribute the ongoing debate and studies aiming at the optimization of the efficacy and application of microbial biostimulants. We propose that joint genomic and phenomic characterization should be routinely included in screening programs and in the development of the technical dossier necessary for registration of the product for marketing. Indeed, an increased knowledge of the possible modes of action and (multi)functional features of a microbial strain can enable data-driven decisions when developing a novel bio-product. Leveraging the information encoded in prokaryotic genomes, it is possible to evaluate the (multi)functional aspects of a candidate strain, that may be confirmed and complemented by the phenotypic observations. We hope that the proposed approach can contribute to the current debate in the European Union and to the establishment of standards for microbial biostimulants, in an effort for removing regulatory bottlenecks that impair their application and development, while promoting their effective adoption by operators.

## Supplementary Information

Below is the link to the electronic supplementary material.

Supplementary Material 1

## Data Availability

Wole genome sequencing data that support the findings of this study have been deposited in NCBI with accession code PRJNA889341 available at: [https://www.ncbi.nlm.nih.gov/bioproject/?term=PRJNA889341](https:/www.ncbi.nlm.nih.gov/bioproject/?term=PRJNA889341). Activity values from phenotype microarray experiment are shared as Supplementary Material.
